# Enhancing Prediction of Brain Tumor Classification Using Images and Numerical Data Features

**DOI:** 10.3390/diagnostics13152544

**Published:** 2023-07-31

**Authors:** Oumaima Saidani, Turki Aljrees, Muhammad Umer, Nazik Alturki, Amal Alshardan, Sardar Waqar Khan, Shtwai Alsubai, Imran Ashraf

**Affiliations:** 1 Department of Information Systems, College of Computer and Information Sciences, Princess Nourah bint Abdulrahman University, Riyadh 11671, Saudi Arabia; ocsaidani@pnu.edu.sa (O.S.); namalturki@pnu.edu.sa (N.A.); amalshardan@pnu.edu.sa (A.A.); 2 Department College of Computer Science and Engineering, University of Hafr Al-Batin, Hafar Al-Batin 39524, Saudi Arabia; tajrees@uhb.edu.sa; 3 Department of Computer Science & Information Technology, The Islamia University of Bahawalpur, Bahawalpur 63100, Pakistan; 4 Department of Computer Science & Information Technology, The University of Lahore, Lahore 54000, Pakistan; sardarwaqarkhan66@gmail.com; 5 Department of Computer Science, College of Computer Engineering and Sciences, Prince Sattam bin Abdulaziz University, Al-Kharj 11942, Saudi Arabia; sa.alsubai@psau.edu.sa; 6 Department of Information and Communication Engineering, Yeungnam University, Gyeongsan 38541, Republic of Korea

**Keywords:** transfer learning, brain tumor prediction, data features, healthcare, MRI images, ensemble learning, UNet, MobileNet

## Abstract

Brain tumors, along with other diseases that harm the neurological system, are a significant contributor to global mortality. Early diagnosis plays a crucial role in effectively treating brain tumors. To distinguish individuals with tumors from those without, this study employs a combination of images and data-based features. In the initial phase, the image dataset is enhanced, followed by the application of a UNet transfer-learning-based model to accurately classify patients as either having tumors or being normal. In the second phase, this research utilizes 13 features in conjunction with a voting classifier. The voting classifier incorporates features extracted from deep convolutional layers and combines stochastic gradient descent with logistic regression to achieve better classification results. The reported accuracy score of 0.99 achieved by both proposed models shows its superior performance. Also, comparing results with other supervised learning algorithms and state-of-the-art models validates its performance.

## 1. Introduction

Rapid advancements in medical imaging techniques have led to significant progress, enabling the swift identification of various diseases. This breakthrough facilitates early intervention, which proves particularly beneficial in cases of life-threatening conditions like cancer, tumors, eye diseases, Alzheimer’s, blood clots, and eye ailments [1]. In diagnosing these severe disorders, biopsies and imaging of the affected regions play a crucial role. Biopsies are utilized to confirm the presence of specific illnesses, while images of the affected areas are commonly employed for early-stage disease detection [2]. In these situations, highly accurate and easily recognizable depictions of the infected areas are crucial for aiding in diagnosis. The brain is one of the important organs of the human body that is essential for managing the body’s organs and making decisions. As a result, brain tumors seriously endanger human life. Many cancers affect the neurological system, particularly the brain parenchyma, and are referred to as metastases [3]. The occurrence rate of brain metastases, which refers to the spread of cancer towards the brain from the rest of the body parts, is ten times higher than that of primary brain tumors [4]. Gliomas represent a specific category of tumors that can exhibit varying degrees of aggressiveness. Pituitary adenomas and meningiomas can be classified from other types of brain tumors.

Primary brain tumors should be diagnosed and treated as soon as possible because of their potential to be fatal and malignant. There are numerous strategies available for managing these malignant tumors, and proper therapy is of the utmost significance. The course of treatment for brain tumors is determined by several variables including when the tumor was diagnosed and what kind it was. Accurate diagnosis of brain tumors can be achieved through the utilization of diagnostic techniques such as magnetic resonance imaging (MRI) [5]. Making timely decisions about treatment alternatives is made easier with the help of MRI, which offers crucial information for tumor classification [6]. Detecting brain tumors at an early stage is of utmost importance in enhancing survival rates and administering timely and suitable treatment. Manual diagnosis and detection methods are often less dependable, time-consuming, labor-intensive, and susceptible to human error. This is where computer-aided imaging technology has made remarkable strides in the realm of medical image analysis.

Among the non-invasive tools available, MRI has emerged as the most widely utilized method for brain tumor detection [7]. MRI scans are frequently used for brain analysis because they can distinguish between soft tissues, giving them an advantage over other methods in the detection of brain tumors. Additionally, since MRI does not expose the brain to ionizing radiation, it has no negative effects [8]. The MRI method is frequently used by radiologists because of its capacity to identify aberrant cell development, including brain tumors. Zahoor et al. [9] introduced a dual-channel brain tumor detection (DC-BTD) system for brain tumor detection. The authors used MRI scans and showed few false negatives. For discriminating, static S-shaped features were used, while the D-channel was used to extract dynamic features. The study made use of four different machine learning classifiers, data normalization, and augmentation. The study’s findings demonstrated greater performance, outperforming earlier research with an accuracy rate of 98.70%. Similar to this, in [10], ensemble models were used to improve MRI images with an average filter to classify and identify brain tumors. ResNet-18 and AlexNet were employed as deep learning models to extract features, and these features were subsequently utilized for classification. The classification was performed using SoftMax and support vector machine (SVM) algorithms. The proposed ensemble model AlexNet+SVM scored 95.10% accuracy. MRI scans were used for the categorization of brain tumors in the investigation by Daz-Pernas et al. [11]. In contrast to earlier investigations, they adopted a method without any pre-processing steps. Despite no preprocessing, the proposed strategy managed to classify tumors with an amazing accuracy rate of 97.3%. It is noteworthy that the majority of imaging methods, including MRI, create grayscale images, but the color Doppler method creates color images. However, alternative techniques have not produced the required outcomes when it comes to tissue segmentation in areas like post-processing [12,13].

Deep learning models have been the subject of numerous studies focusing on brain tumor identification. For example, Khan et al. [7] designed an intelligent deep-learning-based hierarchical deep-learning-based brain tumor (HDL2BT) system to detect brain tumors precisely. The approach divided brain tumors into three groups: gliomas, meningiomas, and pituitary tumors. The proposed model’s excellent precision rate of 92.13% was attained by using convolutional neural networks (CNN). DeepTumorNet [14] developed by Raza et al. also worked on the topic of three types of brain tumor detection. The CNN GoogLeNet architecture served as the framework for the system. With an accuracy score of 98.67%, the researchers had great success evaluating the system using publicly available datasets. These results illustrate the efficiency of deep learning models in correctly classifying and identifying brain tumors. Ahmad et al. conducted a study [15] focusing on deep learning techniques that integrate conventional classifiers with various transfer learning-based deep learning systems for brain tumor detection. They utilized seven transfer learning models in their investigation, InceptionResNetV2, ResNet50, VGG-16, Xception, DenseNet201, VGG-19, and InceptionV3. The study’s results showed a 98.39% accuracy rate for brain tumor detection. This demonstrates how well traditional classifiers and transfer learning can work together to reliably identify brain tumors.

Another subsequent model was proposed by Qureshi et al. [16] for brain tumor detection using a lightweight (computationally less complex) model. The study focused on three types of brain tumors. The proposed system relies on an ultra-lightweight deep learning approach that effectively distinguishes different texture features through the use of a grey-level co-occurrence matrix (GLCM). To detect brain tumors, an HFS is used with an SVM. A maximum accuracy of 99.23% was reported while the F1 score was 99%. The authors discovered a 2% improvement when they compared the study’s findings to those of the most recent system. A technique using min-max normalization on a CNN-based dense EfficientNet was proposed in [17]. The authors proposed an EfficientNet system with dropout layers and dense layers. They combined the min-max with the data augmentation. The authors intended to improve the contrast between cancer cells. In terms of overall performance, the proposed method performs better than any other. The findings revealed that the proposed EfficientNet variation had training and testing accuracy of 99.97% and 98.78%, respectively.

Sharma et al. [18] proposed an improved watershed segmentation technique, which is based on the modified ResNet50, for the precise, accurate, and efficient detection of the tissues associated with brain tumors. Three fully connected layers and five conventional layers were used to implement the ResNet50. They made use of high-dimensional, deep features to achieve the ideal values. The proposed ResNet50-enhanced watershed segmentation scored a 90% accuracy value. Rinesh et al. [19] utilized hyperspectral images in their research to perform various operations for cancer localization in the brain. They employed k-based clustering algorithms, specifically k-means clustering and k-nearest neighbor, to identify the tumor. To determine the appropriate value of k in each experiment, the researchers applied an optimization technique called the firefly method. The different regions of the brain were labeled using a multilayer feedforward neural network. Results show that the proposed method achieved 98.24% specificity, 96.32% sensitivity, and 96.47% accuracy.

Milica et al. [20] conducted research on classifying brain tumors utilizing MRI images. The authors utilized datasets from a medical university and two hospitals for their studies. The researchers employed two databases and a 10-fold cross-validation method two times in the experiments. The study’s findings demonstrated that the proposed CNN with 10-fold cross-validation has a 96.56% accuracy value. A hybrid deep tumor network was proposed by Amran et al. [21] for the classification of brain tumors. In this investigation, the Br35H Kaggle dataset was employed. The GoogleNet architecture combined with a CNN model makes up the proposed ensemble system. The proposed system scored 99.51% accuracy. They also employed various transfer learning models in the study to compare results. For the detection and identification of brain tumors, Naeem Ullah et al. [22] used the publicly accessible dataset. Experimental results demonstrate that the Inceptionresnetv2 transfer learning method has a classification accuracy of 98.91%.

Along the same directions, Hashmi and Osman [23] employed two datasets for tumor classification. An attention technique using an extreme gradient boost and a conditional segmentation approach using a residual network was proposed. Results indicate that the proposed CNN-CRF-Resnet model has the greatest accuracy for the three classes. For the classification of brain tumors, Samee et al. [24] proposed the hybrid transfer learning system GN-AlexNet. They used 10 layers of AlexNet and five layers of GoogleNet in the proposed system. Five transfer learning models were also used. The study’s findings demonstrate that the proposed GN-AlexNet performs better than competing ML/DL models and gets an accuracy rate of 99.51%.

Transfer learning models have been effectively used by several studies for brain tumor detection [21,22]. CNN and GoogleNet were combined by Amran et al. [21] to create a hybrid deep tumor network, which achieved a remarkable accuracy of 98.91%. An accuracy of 98.91% using InceptionResNetV2 is reported in [22]. Similarly, [23] used an attention technique based on extreme gradient boosting (XGB) and a conditional segmentation strategy using a residual network. The CNN-CRFResNet system, derived from this methodology, achieved remarkable accuracy of 99.56% across all three classes.

A deep-learning-based base ensemble model is proposed by Rasool et al. [17] for the efficient classification of the three types of brain tumors. They incorporated a fine-tuned GoogleNet model as part of the ensemble and attained a 93.1% accuracy. However, the authors obtained a higher accuracy of 98.1% when GoogleNet was used as the feature extractor. This highlights the effectiveness of using deep learning models for feature extraction in brain tumor classification tasks. Genomic information can play a crucial role in diagnosing brain cancer by facilitating the classification and segmentation of brain tumors, given that genetic mutations are a significant contributing factor in their development [25]. The combination of artificial intelligence (AI) methods with radio genomics has displayed great potential in identifying brain tumors. This is achieved by utilizing the genomic condition of genetic mutations found in different genes and cellular proteins [26,27]. Consequently, this approach facilitates the recognition of molecular characteristics associated with the disease through radiological medical images [28].

In previous studies, the same dataset used in this study has been employed, yielding commendable results. For instance, Dutta et al. [29] utilized a machine-learning-based approach for the early and accurate detection of brain tumors. The authors compared the performance of the XGBoost classifier with AdaBoost (ADA), gradient boosting classifier (GBC), random forest (RF), and extra-trees (ET) classifiers. Among the employed machine learning models, XGBoost achieved an accuracy of 98.54%, surpassing other models in terms of accuracy. Similarly, the study by Methil et al. [30] presented a deep-learning-based architecture for efficient brain tumor detection, which incorporated various image processing techniques. The authors employed multiple deep learning models and achieved the highest accuracy of 95%. MRI images play a crucial role in brain tumor detection. The study conducted by Shah et al. [31] proposes a system that utilizes brain MRI images. The objective of the research is to identify malignancy in the brain using MRI image data and enhance the accuracy of brain tumor detection. Various image processing techniques and data augmentation methods are used to this end. A maximum accuracy of 98.87% is reported.

This study aims to develop a machine learning and transfer learning model that leverages both image data and numerical features from data to differentiate between patients with brain tumors and those without them. In essence, the proposed system provides the following advantages:

- This study presents a complete framework for the detection of brain tumors using images and feature-based data. The image-based brain tumor detection is utilizing data augmentation techniques and the UNet transfer learning model.
- In this research work, the prediction of brain tumors is performed utilizing CNN features and a voting ensemble model. Stochastic gradient descent and logistic regression classifier are ensembles with soft voting mechanisms to determine the ultimate result.
- The performance of models utilizing convolutional features is compared with that of models that rely on the original features to assess their impact. To perform a performance comparison, this study utilizes a diverse set of machine learning and transfer learning models, including decision tree (DT), Gaussian naive Bayes (GNB), K-nearest neighbor (KNN), random forest (RF), stochastic gradient descent (SGD), logistic regression (LR), extra-trees classifier (ETC), support vector machine (SVM), gradient boosting machine (GBM), and MobileNet models. Furthermore, for comparison, the performance of the proposed system is compared with the various state-of-the-art methods using well-known evaluation parameters, i.e., accuracy, precision, recall, and F1 score.

The rest of the paper is structured as follows. Section 2 presents a detailed discussion of the components and functions of the proposed system, the dataset description, and the supervised learning models used for brain tumor detection. Section 3 presents the results obtained from the experiments. Following that, Section 4 provides discussions on the performance of the proposed approach. Section 5 encompasses the conclusions.

## 2. Materials and Methods

This section describes the ’brain tumor’ dataset that is used for tumor detection, which includes both numerical features and image data. This also provides an overview of the proposed method and outlines the steps involved in the proposed system. Furthermore, it briefly describes the machine learning classifiers and transfer learning techniques that were utilized in this study.

### 2.1. Brain Tumor Dataset

The selection of a suitable dataset plays a crucial role, and for this particular study, the publicly available ‘brain tumor’ dataset from Kaggle is used [32]. For a publicly available dataset, performance validation can be performed by other researchers as well. The prediction of brain tumor detection is performed utilizing twelve features from the dataset. There are a total of 3762 records in the dataset. Among the 12 features, the first five are first order, specifically standard deviation, mean, kurtosis, variance, and skewness, while the remaining eight are texture features including correlation, homogeneity, angular second moment (ASM), entropy, dissimilarity, contrast, energy, and coarseness. The target class is divided into two categories: tumor and non-tumor. Out of the 3762 instances, 2079 belong to the non-tumor class, while 1683 belong to the tumor class. The dataset also includes corresponding images, providing both numerical data and image classification information.

### 2.2. Supervised Learning Algorithms for Brain Tumor Detection

In this study, nine distinct supervised learning algorithms were employed to detect brain tumors. These algorithms comprise DT, k-NN, LR, RF, SGD, ETC, GNB, SVM, and GBM. This section of the study provides a concise overview of each of these machine learning models.

#### 2.2.1. Random Forest

RF is a frequently used machine learning algorithm because of its simplicity, which is built on tree structure [33,34]. Starting with a single random vector, it proceeds sequentially to produce numerous independent random vectors that are dispersed among various trees. As the algorithm advances, the data are divided into child nodes at each node of the tree until it reaches the leaf nodes. Each node separately classifies the objective variables of the features in RF, and a voting mechanism then determines the final classification. RF error can be estimated using the formula below:

(1) $$\begin{array}{c} PE^{*}=P_{\left(i,j\right)}\left(f\left(i,j\right)<0\right) \end{array}$$

The random vectors *i* and *j* serve as graphical representations of probabilities. These random vectors depict the probabilities of different outcomes. The function *f* calculates the average number of votes for the desired outcome from all random vectors [35], and one can compute this number by using the following formula:

(2) $$\begin{array}{c} f\left(i,j\right)=av_{K}I\left(H\left(i\right)=j\right)-max_{y\neq j}av_{K}I\left(h_{k}\left(i\right)=y\right) \end{array}$$

#### 2.2.2. Decision Tree

DT is a renowned machine learning model that models decisions and possible outcomes using a tree-like structure. It can be applied to both regression and classification applications. Each leaf node in DT represents a class label or a numerical value, whereas each inside node reflects a judgment based on a particular characteristic or attribute [36,37]. To build homogeneous subsets of data at each node, the tree is formed by recursively partitioning the data depending on the values of several attributes. For node split, Gini impurity or information gain is used. Once a decision tree has been trained on a labeled dataset, it can be used to generate predictions about new, unforeseen instances by traversing the tree from the root to a leaf node based on the instance’s feature values. Because the learned rules may be represented as a tree structure, decision trees are renowned for their interpretability and simplicity. However, they can be vulnerable to overfitting, particularly if the trees are overly complicated. This problem can be minimized and decision trees’ performance can be enhanced by approaches like trimming and group methods like random forests.

(3) $$\begin{array}{c} Gini=1-\sum_{i=1}^{classes}p{\left(it\right)}^{2} \end{array}$$

#### 2.2.3. K-Nearest Neighbor

KNN is a popular classification method that has been used to study brain tumors and other areas. It does not make any assumptions about the distribution of the data and is regarded as a nonparametric approach [38,39]. Instead, to assign the new data to the class that is closest to the existing classes, KNN examines the similarity between an existing and newly included data point. It can be utilized for recognition and regression issues in addition to classification activities. Due to its inability to draw rapid conclusions from the acquisition of training data, KNN is frequently referred to as a “lazy learner” algorithm.

#### 2.2.4. Logistic Regression

LR is a machine learning classifier that relies on statistics and supervised learning [40,41,42]. It categorizes input qualities (X: input) into different goal values (Y: output). To calculate the probability of falling into class 0 or class 1, LR employs a logistic function. As seen in the equation below, the logistic function is typically depicted as an “S”-shaped curve.

(4) $$f\left(x\right)=\frac{L}{1+e^{-m(v-v_{o})}}$$

To predict probabilities, LR uses the sigmoid function. You can determine the sigmoid function using the formula below.

(5) $$\sigma \left(x\right)=e^{x}\left(e^{x}+1\right),\sigma \left(x\right)=1\left(1+e^{-x}\right)$$

s(x) , the sigmoid function’s output accepts values of 0 or 1, while *X* serves as the input, and the calculation uses the natural logarithm’s base, *e*.

LR is frequently used for binary classification tasks and is especially successful for data that can be separated into linear categories.

#### 2.2.5. Support Vector Machine

SVM is a popular learning algorithm in classification and regression tasks [43]. SVM operates by creating decision boundaries, in the form of hyperplanes, to effectively separate the dataset. A significant advantage of SVM is its capability to handle both linear and nonlinear data efficiently. In cases, where the data can be linearly separated, the hyperplane divides the dataset into two distinct groups. However, when the data are not linearly separable, SVM can leverage a technique called the kernel trick. This technique enables SVM to transform the original input space into a higher-dimensional feature space, where the data can be separated effectively. The transformed coordinates are denoted as x=f(x) , where f(x) represents the feature mapping function.

#### 2.2.6. Gradient Boosting Machine

The key idea behind GBM is to train new trees that can correct the mistakes made by the previous trees in the ensemble [44,45]. At each iteration, GBM identifies the shortcomings of the current ensemble by analyzing the gradients of a loss function concerning the predicted values. The loss function measures the discrepancy between the predicted values and the actual values of the target variable.

(6) y=ax+b+e

where *e* represents the error term.

The loss function quantifies the disparity between the actual and predicted values, thereby providing a measure of the performance of the model on a given dataset. This indicates how well the model captures patterns and makes accurate predictions.

#### 2.2.7. Extra-Trees Classifier

ETC uses the results of various correlated DTs to create the final prediction [46,47]. Using training samples, each DT in the forest is produced and added to the overall classification effort. Using random feature subsets, many uncorrelated DTs are built. The Gini index is used to assess each feature’s quality during tree construction, and feature selection is performed to discover the best way to split the data. An ensemble of DTs is created through this iterative method, and this ensemble makes predictions for the ETC model as a whole.

#### 2.2.8. Gaussian Naive Bayes

GNB’s working is quite simple as during training it learns the probability of each feature as independent of the other feature [48,49]. It is based on the name of the scientist and is well-known as the Bayesian theorem. For tasks involving object classification, this approach is frequently used, especially when the data are evenly distributed. Because of these features, it is known as the GNB classifier.

#### 2.2.9. Stochastic Gradient Decent

Multiple binary classifiers are integrated into SGD, which has undergone thorough testing on sizable datasets [50,51]. It is simple to create and comprehend, and regression approaches are comparable to how it works. It is very important to configure the hyperparameters for SGD accurately to obtain accurate results. Additionally, SGD is sensitive to feature scaling, emphasizing how crucial it is to scale the features correctly before implementing the algorithm.

### 2.3. Feature Engineering

In this study, feature engineering is carried out using a CNN model. The architecture of the CNN utilized in this study comprises four layers: an embedding layer, a flattened layer, a max-pooling layer, and a 1D convolutional layer. The embedding layer has a dimension of 20,000 pixels and incorporates features derived from the brain tumor dataset. The output dimension of the embedding layer is set to 300. Subsequently, a 1D convolutional layer is added, featuring a filter size of 5000, and a kernel size of 2 × 2. The proposed approach utilizes ReLu as an activation function. A max-pooling layer of the size 2 × 2 is added to obtain the desired output from the 1D convolutional features.

To represent the brain tumor dataset, we can denote it as a tuple set ( $fs_{i}$ , $tc_{i}$ ), where $fs_{i}$ represents the feature set of the *i*-th tuple, $tc_{i}$ represents the target class column of the *i*-th tuple, and *I* represents the tuple index. During the training process, the output of the training set was passed through an embedding layer to obtain the desired results.

(7) EL=embedding_layer(Vs,Os,I)

(8) EOs=EL(fs)

**Figure 1:**
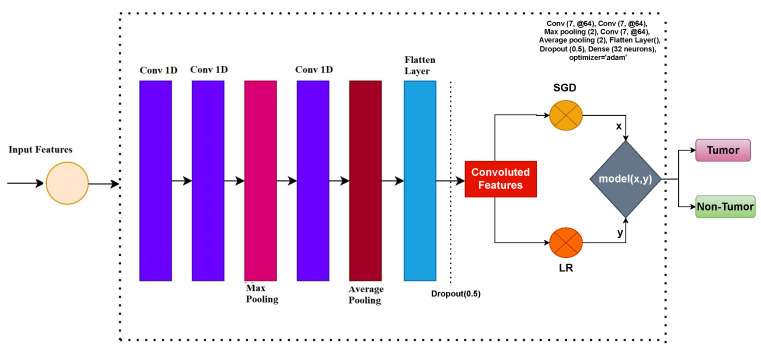
Architecture of proposed voting classifier along with CNN.

In the CNN architecture, the convolutional layer takes the input from the output of the embedding layer, which is denoted as $EO_{s}$ . The embedding layer itself is represented as EL and consists of three parameters: $V_{s}$ (size of the vocabulary), *I* (length of the input), and Os (size of the output). The architecture of the CNN and the predictive model employed in this study is illustrated in Figure 1.

### 2.4. Transfer Learning Models

Transfer learning is a popular technique in machine learning and computer vision that leverages pre-trained models on large datasets to solve new tasks efficiently. In the context of brain tumor classification using image data, two commonly used transfer learning models are U-Net and MobileNet.

#### 2.4.1. U-Net

U-Net, developed by Olaf Ronnenberg et al. in 2015, is a highly influential model in the field of image segmentation. Originally intended for biomedical image segmentation, U-Net quickly gained acclaim for its exceptional accuracy and performance [52]. Notably, it excels at producing impressive results even with limited training data, a common challenge in medical image segmentation. The model is structured around two main paths, resembling an auto-encoder. The first path, known as the contracting or compressive path, acts as the encoder and is constructed using a conventional deep CNN network. The decoder or expanding path (also called the up-sampling or synthesizing path in some publications) comprises both deconvolutional and convolutional layers. The contracting path downsamples input images to diminish their resolution, but the expanding path recovers the original image quality and spatial structure utilizing optimized approaches such as concatenating skip connections. The network learns spatial classification information by providing dense predictions at a greater resolution along the increasing route. It also enhances the resolution of the output image, which is then processed through a final convolutional layer to generate a segmented image with the same dimensions as the input image. Simply put, the network accepts an image with dimensions (h, w, n) and produces an output image that has the same dimensions as of input, where the segmented region corresponds to the area of interest (for example, a brain tumor). This ensures that the shape of the input image remains unchanged throughout the segmentation process. While classification is significant in medical image analysis, it alone cannot provide a pixel-level context representation, as it assigns a single label to the entire image.

U-Net, in conjunction with subsequent optimization methods, was purposely created to handle multi-dimensional tensors, particularly inputs with three or four dimensions. The network produces an output that retains the same shape as the input. Since U-Net’s inception, it has provided a solid foundation for extensive research in medical image segmentation. Numerous advancements have been made by either modifying the original U-Net structure or combining it with other architectures.

#### 2.4.2. Mobilenet

MobileNet is a condensed CNN structure developed to enable efficient processing on devices that have limited capabilities, like mobile phones [53]. It utilizes depthwise-separable convolutions, which greatly decrease the number of parameters and computational complexity while maintaining the effectiveness of the model. MobileNet is frequently employed for diverse image classification assignments due to its efficiency. By employing transfer learning with a pre-trained MobileNet model, the valuable features acquired from extensive image datasets can be utilized to enhance the accuracy of brain tumor classification.

The MobileNet architecture is specifically designed to be efficient and effective, particularly in scenarios where computational resources are limited or when dealing with tasks that require minimal features, such as palmprint recognition. Its notable feature is the depthwise structure, which enables high performance while keeping computational demands to a minimum. The complexity of a 1 × 1 convolution, known as pointwise complexity, is a key consideration. The architecture employs ReLU to preserve pointwise connections while generating deep abstraction layers. Additionally, a resolution multiplier variable denoted as *w* is introduced to reduce the dimensionality of both the input image and the internal representation of each layer. By using this variable, it is possible to alter the network’s dimensions while maintaining a constant value for “*w*” across all layers. By doing this, the model’s overall effectiveness is improved. The feature vector map has a size of Fm , the filter has a size of Fs , and the input variable is indicated by the letter *p*, while the output variable is denoted by the letter *q*. The equation below can be used to evaluate the overall computation efforts for the fundamental abstraction layers of the architecture. The following is an expression for the computation-related work, indicated by the variable ce :

(9) $$c_{e}=F_{s}\cdot F_{s}\cdot w\cdot \alpha F_{m}+w\cdot \rho \cdot \alpha F_{m}\cdot \alpha F_{m}$$

In MobileNet, the multiplier variable *w* has a context-dependent value. It is frequently selected from the range of 1 to n in experimental investigations for the categorization of brain tumors. The resolution multiplier variable *r* is also set to 1. The following equation can be used to evaluate the computational efforts, symbolized by the variable $cost_{e}$

(10) $$cost_{e}=F_{s}\cdot F_{s}\cdot w\cdot \rho \cdot F_{m}\cdot F_{m}$$

### 2.5. Proposed Voting Classifier

**Figure 2:**
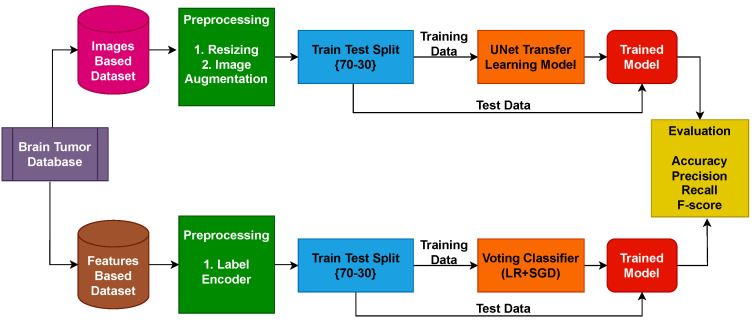
Brain tumor images and feature-based detection framework.

In the previous state-of-the-art research works, different types of machine, deep, and ensemble learning models are applied for brain tumor detection. All of the research works are performed on one type of data either feature-based detection or image-based analysis. None of the previous research work provides complete detection of brain tumors by targeting both types of datasets. Therefore, in this research work, we focused on a two-way brain tumor detection framework. The first phase of this research work focuses on brain tumor detection using image data and the second phase is based on feature-based brain tumor detection techniques. Figure 2 displays the workflow of the pipeline for detecting brain tumors using image-based data.

**Phase 1: Brain Tumor Detection Using Images Analysis**

In the first step of the image-based analysis, all the images of the dataset are converted to the fixed size of 220 × 220. The second step is the augmentation of data to make the dataset balanced as tumor images are less than normal images. The dataset contains 1683 tumor images and 2079 normal images. The augmentation makes both label images 3000. The augmentation hyperparameters are shown in Table 1.

**Figure 3:**
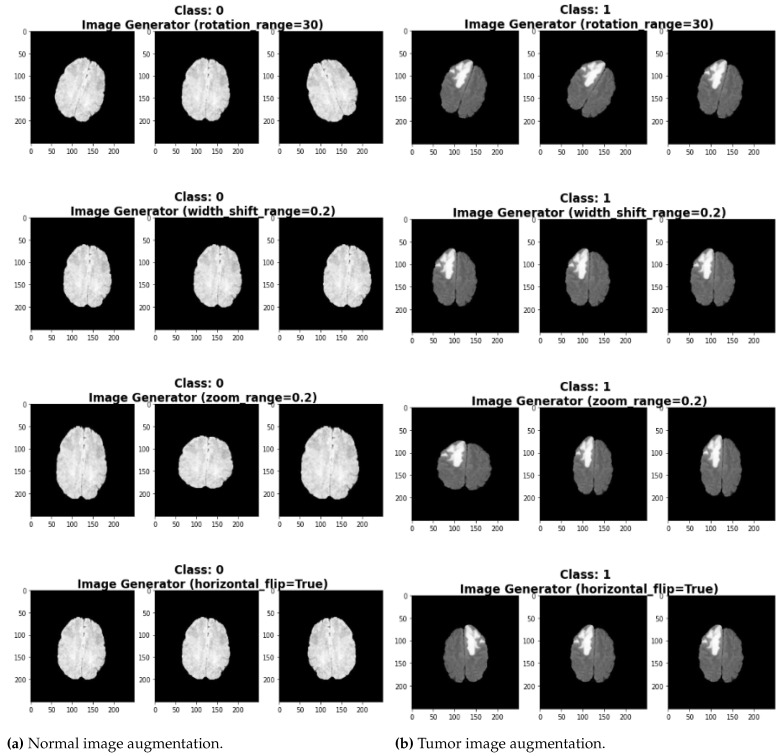
Class-wise data augmentation of images.

The examples of tumor and normal augmented images are shown in Figure 3a,b, respectively. Following augmentation, the images are split into a 70% training set and a 30% testing set to train the UNet model. The model’s performance is evaluated using accuracy, precision, recall, and F1 score.

**Figure 4:**
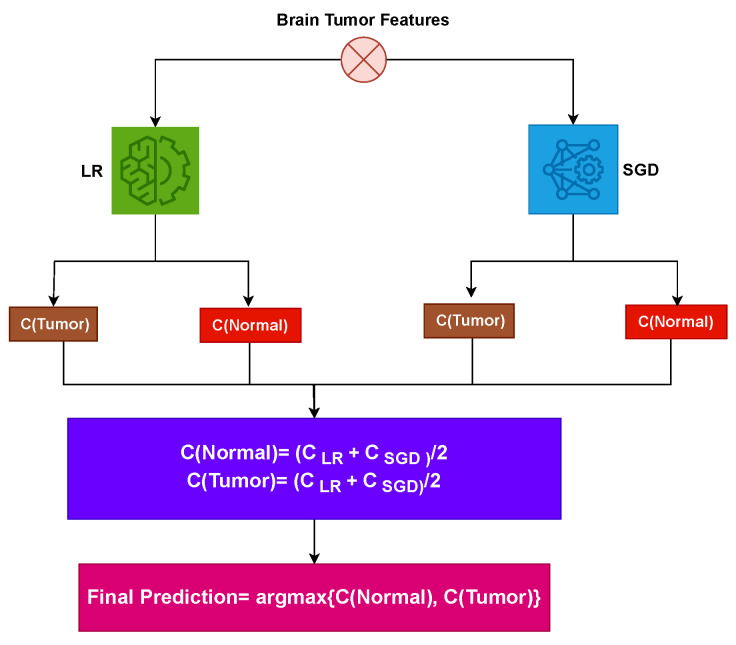
Feature-based brain tumor detection proposed ensemble model.

**Phase 2: Feature-Based Brain Tumor Detection:** Figure 3 shows the workflow of the voting classifier. To accomplish tumor detection, the proposed approach combines LR and SGD. When using the proposed approach, two possibilities are investigated. The first scenario employs all 13 variables of the brain tumor dataset to predict brain tumors. In the second experiment, a CNN is used to extract dataset features. Supervised learning algorithms are employed to classify the tumorous patients among the normal. LR and SGD are joined using soft voting criteria. The architecture of the voting classifier, which implements the soft voting approach, is depicted in Figure 4. In soft voting, the final output is determined by considering the outcome with the highest probability among the combined models.

Soft voting criteria can be represented as

(11) $$\hat{p}=argmax\sum_{i}^{n}LR_{i},\sum_{i}^{n}SGD_{i}$$

The probability values for each test sample in the soft voting technique are given by $\sum_{i}^{n}\mathrm{LR}i$ and $\sum i^{n}{\mathrm{SGD}}_{i}$ , which represent the probabilities assigned by the LR and SGD models, respectively. These probability values are then fed into the soft voting process, as shown in Figure 4, to obtain the final prediction. Each sample that has been processed by the LR and SGD models is assigned a probability score.

### 2.6. Evaluation Metrics

The performance of the trained machine learning models is used to assess their efficacy. Confusion matrix-based evaluation parameters are employed for this purpose. Where TP, TN, FP, and FN stand for true positive, true negative, false positive, and false negative, respectively. For the classification of brain tumors, precision, accuracy, F1 Score, and recall are used. These measures have values between 0 and 1 and are calculated using these equations

(12) $$\begin{array}{c} Accuracy=\frac{TP+TN}{TP+TN+FP+FN} \end{array}$$

(13) $$\begin{array}{c} Precision=\frac{TP}{TP+FP} \end{array}$$

(14) $$\begin{array}{c} Recall=\frac{TP}{TP+FN} \end{array}$$

(15) $$\begin{array}{c} F1score=2\times \frac{Precision\times Recall}{Precision+Recall} \end{array}$$

Accuracy is the proportion of correct predictions (both true positives and true negatives) over the total number of samples in the dataset. In the context of brain tumor detection, it signifies the percentage of correctly classified images, including both correctly identified tumor images and correctly identified normal images.

Precision measures the proportion of true positive predictions (correctly identified tumor images) over the total number of positive predictions (all predicted tumor images). A high precision value indicates that the model has a low false positive rate, meaning that it accurately identifies tumor images without misclassifying normal images as tumors.

Recall (also known as sensitivity or true positive rate), measures the proportion of true positive predictions over the total number of actual positive samples (all actual tumor images). High recall indicates that the model effectively detects most of the positive samples, minimizing false negatives, which are tumors incorrectly classified as normal images.

The F1 score is the harmonic mean of precision and recall. It provides a balanced measure of the model’s performance, considering both false positives and false negatives. A high F1 score indicates a good balance between precision and recall, demonstrating the overall effectiveness of the model in correctly classifying both tumor and normal images.

## 3. Results

The two main scenarios we used in our experiments include using image data and numerical features from data. A CNN is used as the feature engineering method for numerical data. For the image data, two transfer learning models, U-Net and MobileNet, are used.

### 3.1. Experiment Set-up

The performance analysis of the proposed approach involves conducting several experiments and a thorough evaluation of its performance in comparison to alternative learning models. The experiments are conducted on a computer running Windows 10 and equipped with an Intel Core i7 processor from the 7th generation. The implementation of the proposed technique and other learning models is based on TensorFlow, Sci-kit Learn, and Keras libraries in the Python programming language. The experiments are carried out in two different contexts. In the first scenario, numerical features from data are used with CNN as the feature engineering technique. The tumor dataset is employed to extract CNN features. In the second scenario, image data are utilized to train transfer learning models. Table 2 provides the details of experimental setup.

This study uses several machine learning models for performance comparison with the proposed approach. Each model is optimized using hyperparameter fine-tuning to obtain a better performance. A complete list of hyperparameters for all models is provided in Table 3.

### 3.2. Performance of Machine Learning Models Using Featuristic Data

In the first series of experiments, the numerical dataset is used to train the models. It is further divided into two parts. The first part uses CNN as the feature engineering technique, while the second uses the original features.

#### 3.2.1. Performance of Models Using Original Features

Table 4 displays the outcomes of the machine learning models utilizing the numerical dataset. The findings show that SGD and LR have the highest accuracy of 88.1% and 86.9%, respectively, among all the models. The accuracy of RF is 85.4%, whereas the accuracy for the ensemble model LR+SGD is 84.5%. The accuracy of the tree-based ETC model is 82.9%. GNB, on the other hand, performed the worst with an accuracy of 76.9%. It is important to point out that when employing the original feature set, the linear models LR, SGD, and their ensemble outperform other models.

The ensemble model demonstrates notable improvements in performance compared to previous linear models. While LR and SGD individually achieved good results on the original feature set, their combined utilization further enhanced the performance. However, the accuracy of the proposed voting ensemble model does not surpass that of earlier studies. To address this issue, additional experiments are conducted, employing ensemble learning models and CNN as a feature engineering technique. These experiments aimed to enhance the accuracy of brain tumor classification and increase the effectiveness of the categorization process.

#### 3.2.2. Performance of Models Using CNN Features

In order to extract significant features from the dataset, the second set of experiments utilizes CNN features to train the machine learning models. The performance of the proposed ensemble model, as well as other models, is evaluated with the aim of enhancing the feature set and improving accuracy. The results of the machine learning models that utilized CNN as a feature engineering approach are presented in Table 5.

From Table 5, it can be seen that the proposed ensemble model achieves the highest accuracy score of 0.995 among all other models. The ensemble model’s accuracy is significantly higher, surpassing the original feature set by 0.15. Moreover, incorporating CNN features enhances the performance of the various models. The SGD model achieves an accuracy score of 0.987, while the LR model achieves an accuracy score of 0.989. However, the probability-based model GNB performs poorly on the CNN features, with an accuracy score of 0.866. It is noteworthy that GNB outperforms the original features by a significant margin.

#### 3.2.3. Performance of the Transfer Learning Models Using Image Data

In this set of experiments, two transfer learning models, U-Net and MobileNet, are employed. The performance results of these transfer learning models using the image dataset are presented in Table 6.

Results of the transfer learning model shown in the above table depict that the transfer learning model U-Net attained an accuracy score of 99.64% and the MobileNet achieved an accuracy value of 97.28%. Overall, the performance of the transfer learning model is well and outperformed the machine learning model’s performance on the numerical data.

#### 3.2.4. Performance Comparison of Model on Featuristic and Image Data

To evaluate the effectiveness of the transfer learning model (U-Net), we conducted a comparison of the machine learning model and transfer learning models’ performance on the numerical data and the image data. The results clearly demonstrate a substantial improvement in the performance of the transfer learning models, which is 0.14% better than the machine learning model results on CNN features (numerical data) and 15.14% better than the original features (numerical data). Table 7 presents a comprehensive overview of the outcomes obtained by the machine learning models in both scenarios, facilitating a thorough analysis of their performance.

## 4. Discussion

This section provides discussions on the performance of the proposed approach and its limitations.

### 4.1. Performance Comparison with State-of-the-Art Approaches

A comparison with existing state-of-the-art research works is performed to assess the performance of the proposed approach (Table 8). Several recently published articles are chosen to present the most recent developments in the topic. The NGBoost model was used for brain tumor diagnosis in the research by Dutta et al. [29] and achieved an accuracy score of 0.985. Similarly, ref. [30] used the same dataset as the current work and reported an accuracy score of 0.950 using a CNN deep learning model. Other research [31] employed an EfficientNet-B0 model for brain tumor identification and attained an accuracy score of 0.988. In comparison to these existing techniques, the current work is based on an ensemble voting method with CNN features for accurate brain tumor identification, yielding improved results. The proposed model has a classification accuracy of 0.995, showing that it is very accurate and outperforms existing techniques in this domain.

### 4.2. Results of K-fold Cross-validation

For further validation of the proposed approach, k-fold cross-validation is carried out and results are given in Table 9. Results reveal that the proposed approach provides better results with an average accuracy of 99.89%, while per-fold accuracy varies. Similarly, average precision, recall, and F1 scores are 99.52%, 99.49%, and 99.11%, respectively.

### 4.3. Limitations of Study

The proposed two-way brain tumor detection framework combines image-based analysis and feature-based techniques. While data augmentation helps balance the dataset, it may introduce noise, which could impact model performance. Proper selection and fine-tuning of the constituent classifiers (LR and SGD) in the voting classifier are crucial for optimal results. Additionally, the lack of interpretability in deep learning models may raise concerns in medical applications, demanding further research in this aspect.

## 5. Conclusions

This research presents a two-way brain tumor detection framework that combines image-based analysis and feature-based techniques. By integrating the strengths of deep learning models like UNet with the interpretability of traditional classifiers such as LR and SGD, this study has unlocked new avenues for enhanced accuracy and robustness in brain tumor identification and accurately predicting brain tumors in patients with an accuracy of 0.996. Second-phase experimental results indicate that utilizing convolutional features yields superior accuracy compared to using the original features. Additionally, the proposed ensemble classifier surpasses individual models in performance. Comparative analysis with state-of-the-art research demonstrates that the proposed method achieves a higher accuracy score of 0.995, outperforming existing approaches.

This novel approach holds immense potential for early detection and personalized treatment of brain tumors, and its impact extends to transforming medical image analysis and precision medicine. The synergy of cutting-edge technology with traditional methodologies opens exciting possibilities for future exploration, such as transfer learning and ensemble approaches. This work inspires interdisciplinary research in healthcare and highlights the transformative potential of AI in advancing medical practice for a brighter and healthier future.

## Figures and Tables

**Table 1:** Hyper -parameters of data augmentation.

Properties	Value
Rotation range	30
Horizontal flip	‘true’
Zoom range	0.2
Width shift range	0.2

**Table 2:** Experimental setup for the proposed system.

Component	Details
Libraries	Scikitlearn, TensorFlow
Language	Python 3.8
RAM	8 GB
OS	64-bit window 10
CPU	Core i7, 7th Gen with 2.8 GHz processor
GPU	Nvidia, 1060, 8 GB

**Table 3:** Hyperparameter values of all models used in this research work.

Classifiers	Parameters
RF	no. of trees=100, random state=25, max depth=20
DT	no. of trees=100, random state=25, max depth=20
k-NN	algorithm=‘auto’, neighbors=7, leaf size=20, weights=‘uniform’
LR	penalty=‘l2’, solver=‘lbfgs’
SVM	C=2.0, cache size=200, gamma=‘auto’, kernel=‘linear’, maximum iteration=−1, probability=False, random state=25, tol=0.001
GBM	no. of trees=100, random state=25, max depth=20, learning rate=0.1
ETC	no. of trees=100, random state=25, max depth=20,
GNB	alpha=1.0, binarize=0.0
SGD	penalty=‘l2’, loss=‘log’
VC	criteria=‘soft’
CNN	Conv (7, @64), Conv (7, @64), Max pooling (2), Conv (7, @64), Average pooling (2), Flatten Layer(), Dropout (0.5), Dense (32 neurons), optimizer=‘adam’
UNET	Conv (3, @8), Conv (3, @16), Max pooling (2), Conv (3, @128), Average pooling (2), Flatten Layer(), Dropout (0.5), padding (same), optimizer=‘adam’
MobileNET	Conv (3, @8), Conv (3, @16), Max pooling (2), Conv (3, @128), Average pooling (2), Flatten Layer(), Dropout (0.5), padding (same), optimizer=‘adam’

**Table 4:** Brain tumor detection experimental results using original features and machine learning models.

Model	Accuracy	Class	Precision	Recall	F1 Score
Voting Classifier	0.845	Tumor	0.865	0.899	0.878
		Non-Tumor	0.748	0.799	0.776
		Micro Avg.	0.824	0.858	0.856
		Weighted Avg.	0.807	0.843	0.825
GBM	0.805	Tumor	0.795	0.818	0.807
		Non-Tumor	0.818	0.818	0.818
		Micro Avg.	0.805	0.819	0.827
		Weighted Avg.	0.808	0.814	0.826
GNB	0.769	Tumor	0.777	0.788	0.777
		Non-Tumor	0.744	0.766	0.755
		Micro Avg.	0.766	0.777	0.766
		Weighted Avg.	0.766	0.777	0.766
ETC	0.829	Tumor	0.806	0.806	0.806
		Non-Tumor	0.815	0.815	0.815
		Micro Avg.	0.805	0.805	0.805
		Weighted Avg.	0.809	0.820	0.811
LR	0.869	Tumor	0.866	0.899	0.877
		Non-Tumor	0.888	0.899	0.888
		M Avg.	0.855	0.902	0.883
		W Avg.	0.855	0.884	0.876
SGD	0.881	Tumor	0.903	0.892	0.893
		Non-Tumor	0.923	0.924	0.922
		Micro Avg.	0.922	0.922	0.911
		Weighted Avg.	0.919	0.919	0.919
RF	0.854	Tumor	0.827	0.858	0.834
		Non-Tumor	0.844	0.806	0.828
		Micro Avg.	0.844	0.844	0.833
		Weighted Avg.	0.833	0.833	0.833
DT	0.829	Tumor	0.806	0.822	0.811
		Non-Tumor	0.805	0.833	0.814
		Micro Avg.	0.807	0.809	0.818
		Weighted Avg.	0.818	0.804	0.804
SVM	0.788	Tumor	0.788	0.800	0.799
		Non-Tumor	0.777	0.788	0.788
		Micro Avg.	0.788	0.799	0.800
		Weighted Avg.	0.788	0.799	0.800
KNN	0.828	Tumor	0.788	0.822	0.800
		Non-Tumor	0.777	0.811	0.800
		Micro Avg.	0.777	0.811	0.800
		Weighted Avg.	0.799	0.824	0.824

**Table 5:** Rain tumor detection experimental results using CNN features and machine learning models.

Model	Accuracy	Class	Precision	Recall	F1 score
Voting Classifier	0.995	Tumor	0.999	0.999	0.999
		Non-Tumor	0.999	0.999	0.999
		Micro Avg.	0.999	0.999	0.999
		Weighted Avg.	0.999	0.999	0.999
GBM	0.905	Tumor	0.928	0.944	0.926
		Non-Tumor	0.915	0.923	0.914
		Micro Avg.	0.927	0.931	0.924
		Weighted Avg.	0.915	0.935	0.918
GNB	0.866	Tumor	0.877	0.888	0.877
		Non-Tumor	0.844	0.866	0.855
		Micro Avg.	0.866	0.877	0.877
		Weighted Avg.	0.855	0.877	0.866
ETC	0.926	Tumor	0.907	0.903	0.905
		Non-Tumor	0.914	0.918	0.914
		Micro Avg.	0.913	0.913	0.913
		Weighted Avg.	0.900	0.900	0.900
LR	0.989	Tumor	0.966	0.999	0.977
		Non-Tumor	0.988	0.999	0.988
		M Avg.	0.977	0.999	0.988
		W Avg.	0.977	0.999	0.988
SGD	0.987	Tumor	0.985	0.997	0.986
		Non-Tumor	0.999	0.986	0.988
		Micro Avg.	0.988	0.988	0.988
		Weighted Avg.	0.988	0.988	0.988
RF	0.958	Tumor	0.927	0.954	0.935
		Non-Tumor	0.944	0.960	0.952
		Micro Avg.	0.944	0.960	0.952
		Weighted Avg.	0.934	0.954	0.944
DT	0.936	Tumor	0.900	0.928	0.914
		Non-Tumor	0.900	0.934	0.912
		Micro Avg.	0.900	0.900	0.915
		Weighted Avg.	0.914	0.900	0.900
SVM	0.978	Tumor	0.974	0.922	0.955
		Non-Tumor	0.977	0.944	0.944
		Micro Avg.	0.977	0.933	0.944
		Weighted Avg.	0.988	0.955	0.966
KNN	0.982	Tumor	0.988	0.988	0.988
		Non-Tumor	0.977	0.977	0.977
		Micro Avg.	0.966	0.966	0.966
		Weighted Avg.	0.977	0.977	0.977

**Table 6:** Performance of transfer learning models.

Model	Accuracy	Precision	Recall	F1 score
U-Net	99.64%	99%	98%	98%
MobileNet	97.28%	97%	97%	97%

**Table 7:** Performance analysis of feature-based and image-based techniques.

Numerical Features from Data			Image Data	
**Machine Learning Models’ Results**			**Transfer Learning Models’ Results**	
**Models**	**Original Features**	**CNN Features**	**Models**	**Accuracy**
VC (LR+SGD)	84.5%	99.5%	U-Net	99.64%
SGD	88.1%	98.7%	MobileNet	97.28%

**Table 8:** Brain tumor detection performance comparison of previous studies.

Reference	Year	Approach	Accuracy
[29]	2020	NGBoost	0.985
[30]	2021	CNN	0.950
[31]	2022	EfficientNet-B0	0.988
Proposed	2023	Ensemble learning with CNN features	0.995
Proposed	2023	UNet model with data augmentation	0.996

**Table 9:** Results of 5-fold cross-validation.

Model	Accuracy	Precision	Recall	F1 score
1st fold	99.52	99.13	99.61	99.12
2nd fold	99.25	98.34	99.74	99.23
3rd fold	99.64	99.67	99.98	99.81
4th fold	99.08	99.78	99.99	99.85
5th fold	99.98	99.15	99.86	99.33
**Average **	**99.89**	**99.52**	**99.49**	**99.11**

## Data Availability

The dataset utilized in this research is publicly available and can also be requested from the authors.
